# An Improved Artificial Lemming Algorithm and Its Preliminary Application to NIR-Based Prediction of *Dendrobium huoshanense* Polysaccharides

**DOI:** 10.3390/biomimetics11080590

**Published:** 2026-08-18

**Authors:** Yu Liu, Feilong Yu, Yaqi Yang, Xingyu Gao, Maosheng Fu, Chaochuan Jia, Zhengyu Liu

**Affiliations:** 1 School of Electronic Information and Artificial Intelligence, West Anhui University, Lu’an 237012, China; 2 Anhui Province Intelligent Hydraulic Machinery Joint Construction Subject Key Laboratory, Lu’an 237012, China

**Keywords:** *Dendrobium huoshanense* polysaccharide, near-infrared spectroscopy, BP neural network, IALA, periodic mutation, content prediction

## Abstract

*Dendrobium* polysaccharide is an important indicator for evaluating the quality of *Dendrobium huoshanense*. To improve the prediction accuracy of polysaccharide content, this study proposes an improved Artificial Lemming Algorithm (IALA) optimized BP neural network model. In IALA, a periodic mutation strategy and a fast hybrid opposition learning strategy (FHOBL) are introduced to enhance population diversity, improve global search ability, and avoid premature convergence. The proposed IALA was first evaluated on CEC2017 and CEC2020 benchmark functions. Experimental results show that IALA achieves better or competitive performance compared with seven other algorithms in terms of mean fitness, best fitness, and standard deviation. Statistical tests, including Wilcoxon rank-sum and Friedman tests, further verify the significant superiority and robustness of IALA. Then, IALA was used to optimize the initial weights and thresholds of BP neural networks for *Dendrobium* polysaccharide content prediction. The results show that IALA-BP achieves the best overall prediction performance, with an *R*^2^ of 0.8731, RMSE of 2.1581, and MSE of 4.6683. Compared with standard BP and other optimized BP models, IALA-BP provides more accurate and stable prediction results. Therefore, the proposed IALA-BP model is effective for rapid prediction of *Dendrobium* polysaccharide content.

## 1. Introduction

*Dendrobium huoshanense* is one of the most valuable medicinal herbs in traditional Chinese medicine owing to its remarkable pharmacological activities, including antioxidant, immunomodulatory, anti-inflammatory, anti-fatigue, and hypoglycemic effects [1,2,3]. With the rapid expansion of the functional food and herbal medicine industries, the demand for reliable quality evaluation of *Dendrobium huoshanense* has increased substantially [4]. Among its numerous bioactive constituents, polysaccharides are widely recognized as the principal active compounds and serve as one of the most important quality indicators for evaluating the medicinal efficacy and commercial value of *Dendrobium* [5,6]. Therefore, the rapid and accurate determination of polysaccharide content is of great significance for cultivation management, harvesting, processing, and quality control.

Conventional analytical methods for polysaccharide determination, such as the phenol–sulfuric acid assay and high-performance liquid chromatography, provide satisfactory analytical accuracy but generally require complicated sample preparation, expensive reagents, professional laboratory instruments, and time-consuming experimental procedures [7,8,9]. These disadvantages limit their application in rapid and large-scale quality inspection. Near-infrared spectroscopy (NIRS) has emerged as an efficient alternative because of its rapid analysis speed, nondestructive nature, low operating cost, and minimal sample preparation requirements [10,11,12]. Nevertheless, NIR spectra usually exhibit high dimensionality, severe spectral overlap, strong variable correlation, and nonlinear relationships with chemical constituents, making accurate quantitative modeling a challenging task [13].

To address these challenges, chemometric and machine learning methods have been extensively introduced into spectroscopic analysis. Traditional linear models, such as partial least squares regression (PLSR), have achieved satisfactory performance in many applications; however, they often fail to capture the complex nonlinear relationship between spectral variables and polysaccharide content [10]. Artificial neural networks, particularly the back propagation (BP) neural network, possess excellent nonlinear mapping capability and have therefore been widely applied in quantitative spectral analysis [14]. Despite these advantages, conventional BP networks are highly dependent on the initialization of weights and thresholds, exhibit slow convergence, and are prone to becoming trapped in local optima, which may lead to unstable prediction performance and poor generalization ability [15].

Recently, swarm intelligence optimization algorithms have been widely employed to optimize neural network parameters because of their excellent global search capability. Compared with conventional optimization approaches, swarm intelligence algorithms can effectively improve the initialization of BP neural networks, accelerate convergence, and enhance prediction accuracy [16,17,18]. However, many existing optimization algorithms still suffer from premature convergence, insufficient population diversity, and reduced search capability during the later stages of optimization, particularly when dealing with complex nonlinear optimization problems [19,20]. These limitations motivate the development of more efficient optimization strategies with stronger exploration and exploitation abilities.

To overcome these shortcomings, this study employs an Improved Artificial Lemming Algorithm (IALA) to optimize the weights and thresholds of the BP neural network. The proposed IALA incorporates multiple improvement strategies to enhance population diversity, strengthen global exploration capability, and improve local exploitation performance, thereby effectively avoiding premature convergence and improving optimization accuracy. By integrating IALA with BP, the proposed IALA-BP model is expected to achieve higher prediction accuracy, faster convergence, and stronger robustness than the conventional BP model in predicting *Dendrobium* polysaccharide content.

Therefore, this study proposes a rapid and nondestructive quantitative analysis method for *Dendrobium* polysaccharide content based on near-infrared spectroscopy and the IALA-BP neural network. Appropriate spectral preprocessing and feature extraction techniques are first employed to improve spectral quality, followed by the construction of the IALA-BP prediction model. The proposed method is systematically evaluated using multiple statistical indicators and compared with conventional BP and other representative machine learning models. The developed framework provides an accurate, efficient, and intelligent solution for the quality evaluation of *Dendrobium* while further demonstrating the potential of improved swarm intelligence algorithms in spectroscopic quantitative analysis.

## 2. Materials and Methods

### 2.1. Near-Infrared Spectral Data and Polysaccharide Content Acquisition

The *Dendrobium huoshanense* samples used in this study were collected from Huoshan County, Anhui Province, originating from two distinct growth environments—under-forest and rocky habitats—with a total of 157 samples. Near-infrared (NIR) spectral data were acquired using a handheld NIR-R210 spectrometer; leaves were removed prior to data collection, retaining only the stem segments, and the spectral scanning range was 900–1700 nm.

In this study, the classic phenol–sulfuric acid method was employed to determine the polysaccharide content of *Dendrobium huoshanense*, serving as the output target for a back-propagation (BP) neural network model. Briefly, crude polysaccharides were first extracted from dried samples using hot water extraction and ethanol precipitation; subsequently, a standard curve was constructed using *D*-glucose as the reference standard, and the absorbance of the reaction system was measured at 490 nm using a UV–Vis spectrophotometer; finally, the precise polysaccharide content was calculated based on the standard curve equation. These measured values served as labels to guide the training of the BP neural network and to validate its prediction accuracy.

### 2.2. Improved Artificial Lemming Algorithm

The Artificial Lemming Algorithm, proposed by Xiao et al., belongs to a new generation of bio-inspired intelligent optimization algorithms [21]. Modeled on the collective survival behaviors of Arctic lemmings, the algorithm treats each lemming as a candidate solution to an optimization problem; by mathematically modeling four distinct biological behaviors, it automatically balances global exploration and local exploitation, effectively mitigating issues such as premature convergence and poor robustness in high-dimensional optimization often found in traditional algorithms. An improved IALA based on periodic variation and a lightweight hybrid reverse learning strategy is proposed, which effectively enhances the global exploitation and local exploration capabilities.

#### 2.2.1. Initialization

IALA generates the population using random initialization. Let *N* be the population size and *d* be the problem dimension; the position of the *i*-th lemming is then represented by Equation (1).

(1) $$X_{i}=[x_{i1},x_{i2},\cdots ,x_{id}],i=1,2,\ldots ,N$$

Variables for each dimension are randomly generated according to a uniform distribution, as shown in Equation (2).

(2) $$x_{ij}=L_{j}+r\cdot (U_{j}-L_{j})$$

*D*_j_ and *U*_j_ represent the lower and upper bounds of the *j*-th variable, respectively, and *r* is a uniformly distributed random number between 0 and 1.

#### 2.2.2. Core Biomimetic Behaviors

The IALA categorizes four types of behaviors into two major stages; it switches search modes based on random probabilities and introduces an energy decay coefficient *E* that gradually decreases with iterations, thereby enabling a transition from global, large-scale searching to local, refined optimization.

Long-distance migration behavior is simulated by combining Brownian motion and optimal individual guidance to realize large-scale traversal of the search space. The position update formula is shown as Equations (3) and (4).

(3) $${\vec{X}}_{i}(t+1)={\vec{X}}_{b}(t)+F\times \vec{B}\times (\vec{r}\times \left({\vec{X}}_{b}(t)-{\vec{X}}_{i}(t)\right)+(1-\vec{r})\times \left({\vec{X}}_{i}(t)-{\vec{X}}_{a}(t)\right))$$

(4) $$\vec{r}=2\times \mathrm{rand}(1,d)-1$$

where ${\vec{X}}_{b}(t)$ is the global optimal position of the population at the *t*-th iteration; $\vec{B}$ is the Brownian motion vector obeying standard normal distribution; ${\vec{X}}_{a}(t)$ represents the position of a random individual in the current population.

Lemmings dig underground tunnels to develop new living areas, which enables individuals to break through the current local search domain. The burrowing operator perturbs individual positions in a wide range based on the search space boundary, and the update rule is shown as Equations (5) and (6).

(5) $${\vec{X}}_{i}(t+1)={\vec{X}}_{i}(t)+F\times D\times ({\vec{X}}_{b}(t)-{\vec{X}}_{a}(t))$$

(6) $$D=r\times (1+sin(\frac{t}{2}))$$

where *L* is a random number related to the current iteration number.

Foraging behavior corresponds to a local, refined search in the vicinity of the global optimum. A spiral search strategy is introduced to enhance local exploitation capabilities; the position update formulas are given by Equations (7) and (8).

(7) $${\vec{X}}_{i}(t+1)={\vec{X}}_{b}(t)+F\times s\times r\times {\vec{X}}_{i}(t)$$

(8) $$s=\sqrt{\sum_{j=1}^{d}{\left({\vec{X}}_{b}(t)-{\vec{X}}_{i}(t)\right)}^{2}}\times (sin(2\pi \times r)+cos(2\pi \times r))$$

When facing natural predators, lemmings make rapid, localized positional adjustments to avoid danger. This operator employs Lévy flights to perturb high-quality individuals, thereby effectively exploring the neighborhood of the optimal solution and enhancing convergence accuracy; the corresponding update formulas are presented in Equations (9) and (10).

(9) $${\vec{X}}_{i}(t+1)={\vec{X}}_{b}(t)+2\times F\times (1-t/T_{\max})\times Levy(d)\times ({\vec{X}}_{b}(t)-{\vec{X}}_{i}(t))$$

(10) $$Levy(d)=0.01\times \frac{u\times \sigma }{{\left|\nu \right|}^{\frac{1}{\beta }}},\sigma ={\left(\frac{\Gamma (1+\beta )\times sin(\frac{\pi \beta }{2})}{\Gamma (\frac{1+\beta }{2})\times \beta \times 2^{\left(\frac{\beta -1}{2}\right)}}\right)}^{\frac{1}{\beta }}$$

where Levy(*d*) represents the *d*-dimensional Lévy flight random vector with a step size obeying the Lévy distribution.

#### 2.2.3. Periodic Mutation Mechanism

To address the issues of premature convergence and the degradation of population diversity encountered by ALA during the late stages of optimization, IALA introduces a periodic mutation strategy characterized by a fixed oscillation period and random amplitude perturbations into the optimization framework [22]. This intermittent mutation pattern effectively perturbs elite individuals that have become trapped in stagnation and explores potential optimal regions outside the current search space, thereby significantly enhancing the global exploration capability of the ALA. The trigger condition for mutation is determined by the modulo operation on the current iteration count, as expressed in Equation (11).

(11) $${\vec{X}}_{i}^{\prime }(t)=\left\{\begin{array}{ll} {\vec{X}}_{i}(t)⊙\left(1+(0.5-\vec{rand})\right), & \mathrm{mod}(t,AT)=0\\ {\vec{X}}_{i}(t), & \mathrm{otherwise} \end{array}\right.$$

where ${\vec{X}}_{i}^{\prime }(t)$ represent the mutated position of the *i*-th individual; **1** denotes the *d*-dimensional all-one vector; $\vec{rand}$ is a *d*-dimensional random vector uniformly distributed in [0, 1].

#### 2.2.4. FHOBL

To enhance the exploration and exploitation capabilities of the algorithm while maintaining computational efficiency, a lightweight hybrid enhancement operator—the Fast Hybrid Opposition-Based Learning (FHOBL) operator—is proposed. This operator integrates local elite-aware adaptive opposition-based learning (OBL) with a simplified differential evolution (DE) perturbation mechanism within a single evaluation budget, thereby significantly reducing computational overhead. First, a local search boundary is constructed based on population dynamics, as shown in Equation (12).

(12) $$L_{loc}=max(min(X),L_{j}),U_{loc}=min(max(X),U_{j})$$

A convergence metric *ρ* is further introduced, as shown in Equation (13).

(13) $$\rho =\frac{max(f)-min(f)}{max(f)+\varepsilon }$$

where ***f*** being the fitness vector and *ε* = 10^−8^, each individual is assigned one of three strategies as shown in Equations (14) to (16).

(1) Local-refracted OBL: For poorly performing individuals, a refracted opposite point is generated as Equation (14).

(14) $$X^{cand}=k\cdot (L_{loc}+U_{loc}-X_{i})+(1-k)X_{i}$$

where k = 0.8 − 0.2*ρ* + (0.2*ρ*)⋅*rand*(0, 1) adapts to the search stage.

(2) Simplified DE/current-to-best/1: A computationally efficient DE variant updates selected individuals via Equation (15).

(15) $$X^{cand}=X_{i}+R(X_{b}-X_{i})+R(X_{r1}-X_{r2})$$

where *R* = 0.5 + 0.5⋅*rand*(0, 1), and *r*_1_, *r*_2_ are distinct random indices.

(3) Adaptive Gaussian perturbation: To escape stagnation, a local Gaussian noise is injected by Equation (16).

(16) $$X^{cand}=X_{i}+N(0,\sigma^{2}I),\sigma =0.05(1-\rho )(U_{loc}-L_{loc})$$

The feasibility of all candidate solutions is ensured through boundary repair, as shown in Equation (17).

(17) $$X_{j}^{cand}=\left\{\begin{array}{c} L_{j},\;\;if\;\;\;\;\;\;X_{j}^{cand}<L_{j}\\ U_{j},\;\;if\;\;\;\;\;\;X_{j}^{cand}>U_{j}\\ X_{j}^{cand},\;\;otherwise \end{array}\right.$$

Critically, only one new candidate per individual is evaluated per generation, ensuring the total number of objective function calls remains *N*, i.e., *O*(*N*) time complexity.

#### 2.2.5. Exploration–Exploitation Transition Mechanism

To achieve a dynamic transition between global exploration and local exploitation, IALA introduces a mode transition control factor, *E*, as shown in Equation (18).

(18) $$E(t)=4\times \arctan\left(1-\frac{t}{T}\right)\times \ln\left(\frac{1}{rand}\right)$$

Here, *t* represents the current iteration number, and *T* is the maximum number of iterations. As the number of iterations increases, *E* gradually decreases, enabling the algorithm to transition from large-scale search in the early stages to refined exploitation in the later stages. When E > 1, the algorithm executes a global exploration strategy to enhance population diversity; when *E* < 1, it enters the local exploitation phase to improve convergence speed and optimization accuracy. Through this control factor, IALA can adaptively adjust its search mode across different stages, thereby achieving a better balance between exploration and exploitation capabilities.

#### 2.2.6. Flowchart of IALA

**Figure 1:**
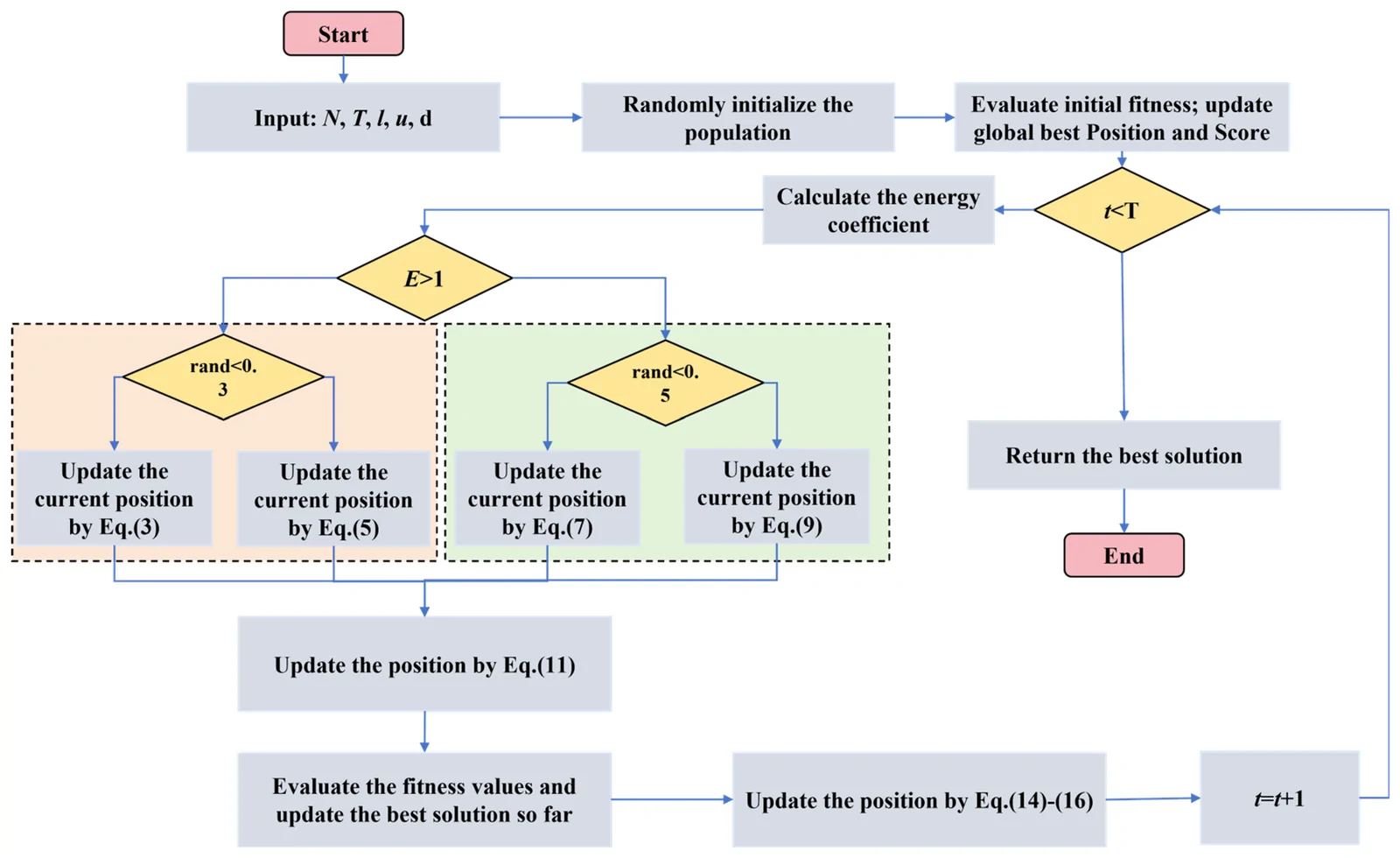
The flowchart of IALA.

Figure 1 presents the flowchart of IALA. Based on the flowchart, the algorithm first receives the input parameters. Then, the population is randomly initialized. The fitness values of all individuals are evaluated, and the initial best position and best score are recorded.

During the iterative process, the algorithm first checks whether the current iteration satisfies *t* < T. If so, the energy coefficient is calculated to control the transition between exploration and exploitation. When *E* > 1, the algorithm enters the global exploration stage. According to the condition *rand* < 0.3, the position of each individual is updated by Equation (3) or Equation (5), which helps enhance population diversity and global search ability. When *E* < 1, the algorithm enters the local exploitation stage. According to the condition *rand* < 0.5, Equation (7) or Equation (9) is used to update the current position, thereby improving local search accuracy and convergence performance.

After that, the position is further updated by Equation (11). Then, the fitness values are recalculated, and the best solution found so far is updated. Subsequently, the position is further optimized by Equations (14)–(16), and the iteration counter is updated as t = t + 1. This process is repeated until the maximum iteration number is reached. Finally, the algorithm returns the best solution.

### 2.3. BP Neural Network Based on IALA

**Figure 2:**
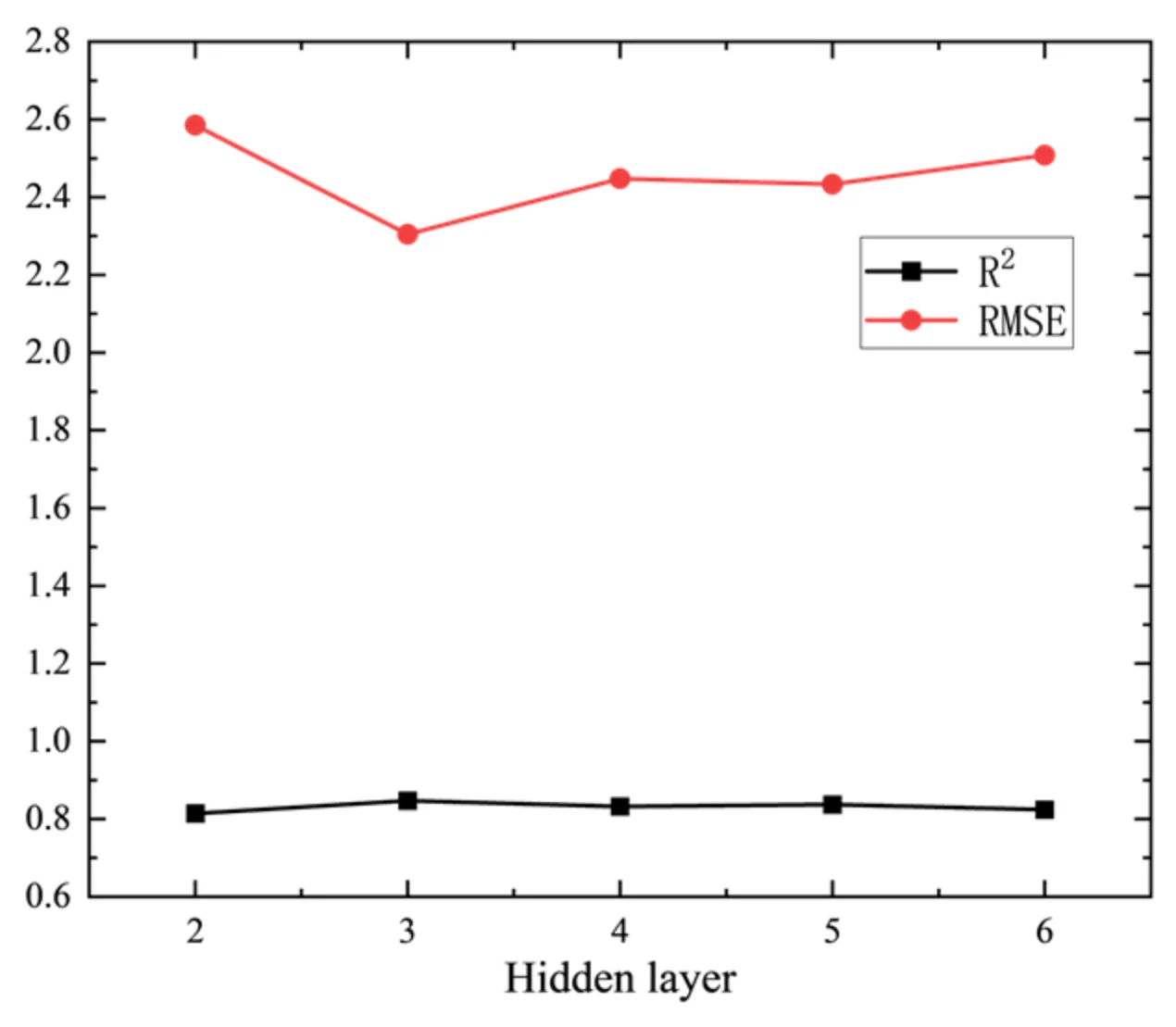
Sensitivity analysis of the BP neural network under different numbers of hidden-layer neurons.

To enhance the generalization capability and training stability of the BP neural network, this paper employs IALA to globally optimize the network’s initial weights and thresholds. The original dataset is partitioned into training and testing sets at a 3:1 ratio. A single-hidden-layer BP network is constructed, with the number of nodes in the input, hidden, and output layers determined by the feature dimensionality, the preset number of hidden units, and the dimensionality of the target variable, respectively. To determine the appropriate number of hidden-layer neurons, a sensitivity analysis was conducted using 2–6 neurons under identical experimental settings. As shown in Figure 2, the network with three hidden neurons achieved the highest R^2^ and the lowest RMSE among all tested architectures. When only two neurons were used, the relatively low R^2^ and high RMSE indicated insufficient nonlinear fitting capacity. Increasing the number of neurons from three to six did not further improve prediction performance; instead, R^2^ decreased slightly and RMSE increased. These results indicate that additional network complexity did not provide a generalization benefit for the present dataset. Therefore, three hidden-layer neurons were selected as the optimal architecture, providing the best balance between prediction accuracy and model complexity.

The maximum number of BP training epochs was 1000, and the performance goal was 1 × 10^−6^. The remaining Levenberg–Marquardt parameters were retained at their MATLAB R2024a defaults, including an initial damping factor of 0.001, a damping decrease factor of 0.1, a damping increase factor of 10, a maximum damping factor of 1 × 10^−10^, a minimum gradient of 1 × 10^−7^, and a maximum of six validation failures. Training terminated when any applicable stopping condition was reached. The IALA is employed for optimization with a population size of 5, a maximum of 30 iterations, and search boundaries of [−1.35, 1.35]; the fitness function is defined as the mean squared error (RMSE) of the BP network on the training set. Upon obtaining the optimal weight-threshold vector z* through the IALA global search, the vector is decoded according to the network structure and assigned to the input weights, hidden layer thresholds, output weights, and output thresholds, thereby completing the network’s initial configuration.

### 2.4. Ablation Experiment

**Figure 3:**
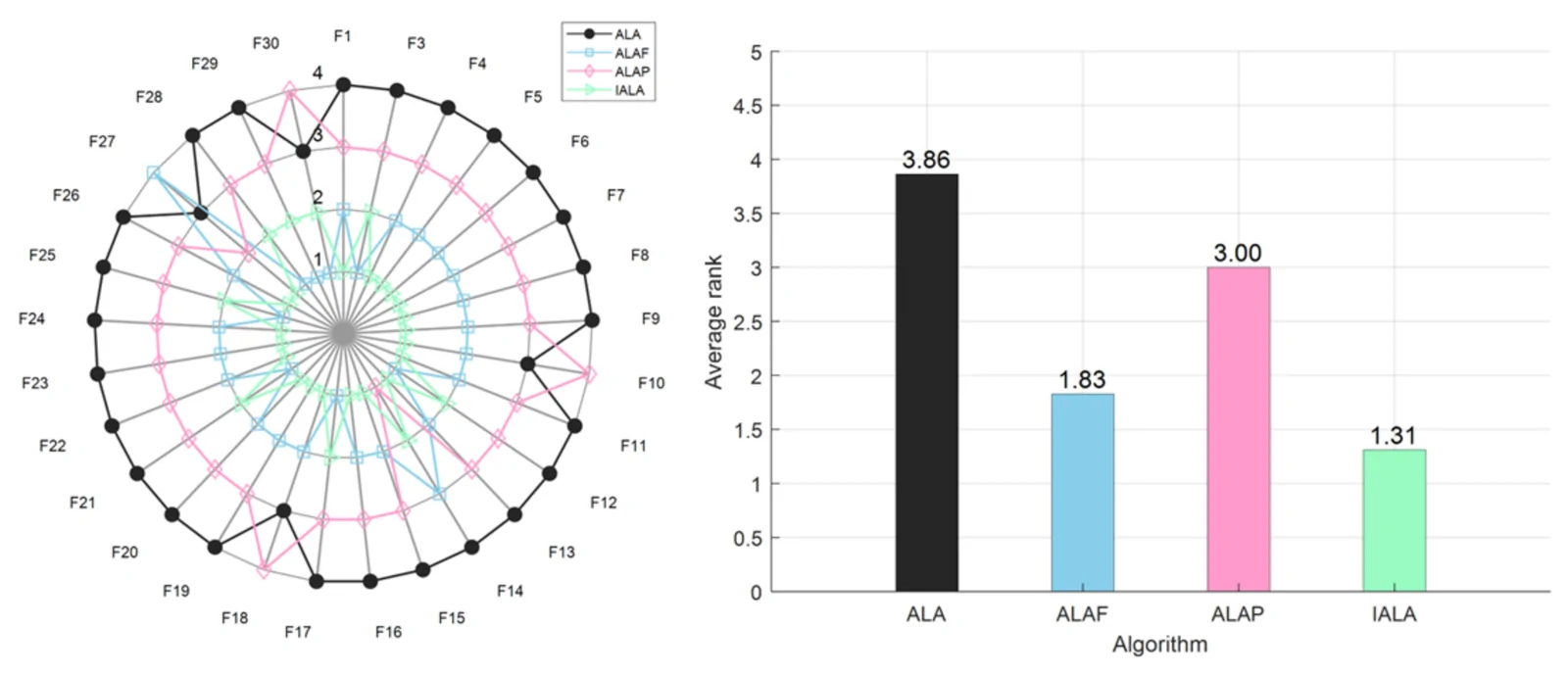
Radar and ranking charts for CEC2017 (Ablation Experiment).

Figure 3 presents the ablation results on the CEC2017 benchmark functions. A lower average rank indicates better overall performance. The original ALA obtained an average rank of 3.86, whereas ALAP (Periodic Mutation) and ALAF (FHOBL) achieved improved ranks of 3.00 and 1.83, respectively. These results demonstrate that both Periodic Mutation and FHOBL contribute positively to the optimization performance of ALA, with FHOBL providing the larger individual improvement. The complete IALA achieved the best average rank of 1.31, outperforming both single-strategy variants. This result indicates that Periodic Mutation and FHOBL are complementary: Periodic Mutation helps maintain population diversity and escape local optima, while FHOBL strengthens search efficiency and solution refinement. Their integration therefore provides better overall performance than either strategy alone.

## 3. Performance Evaluation of IALA

### 3.1. Experimental Setup

The simulation platform runs on a Windows 11-based computer equipped with an Intel® Core™ i9-14900KF processor (Intel Corporation, Santa Clara, CA, USA), 32 GB of RAM, and an NVIDIA GeForce RTX 5080 graphics card (NVIDIA Corporation, Santa Clara, CA, USA). All algorithms are implemented within the MATLAB R2024a environment. The CEC2017 and CEC2020 test suites were used to evaluate algorithm performance, with dimensions of 30, 50, and 100 selected for both [23,24]. Furthermore, the results for 30-dimensional cases from the CEC2017 and CEC2020 standard test functions were selected for detailed analysis. Seven algorithms were chosen for comparison—ALA, LSHADE [25], Particle Swarm Optimization (PSO) [26], Stochastic Social Learning Optimization (SSLO) [27], Differential Evolution Algorithm (DE) [28], Undivided Family Interaction Algorithm (UFIA) [29], and Grey Wolf Optimizer (GWO) [30]—encompassing classic, championship-winning, and state-of-the-art algorithms. All algorithms employ the same parameter settings: a population size of 20 and a maximum of 1000 iterations.

### 3.2. Overall Performance Comparison

Table 1 presents the average fitness values of all comparative algorithms on the 30-dimensional CEC2017 benchmark suite. Overall, the proposed IALA achieves competitive performance across the 30 test functions and obtains the best results on the majority of benchmark problems, 17 out of 29 test functions achieved first place. Compared with ALA, IALA consistently produces lower mean fitness values on most functions, demonstrating that the proposed periodic mutation and FHOBL strategies effectively improve the optimization capability of ALA.

When compared with LSHADE, PSO, DE, SSLO, UFIA and GWO, IALA obtains the optimal or suboptimal mean fitness on most 30-dimensional benchmark problems. The periodic mutation module periodically disturbs individuals to avoid premature convergence, while the FHOBL strategy strengthens local exploitation around elite solutions. Their synergy enables IALA to balance exploration and exploitation well, and presents stronger stability when facing high-dimensional complex optimization landscapes. The comparative results fully demonstrate the effectiveness of the hybrid improvement strategies embedded in IALA.

Table 2 reports the best fitness values obtained by all compared algorithms on the CEC2017 benchmark suite. As shown in Table 2, the proposed IALA achieves the best results on most benchmark functions, demonstrating its strong capability in discovering high-quality solutions. Compared with ALA, IALA obtains lower minimum fitness values on most functions, indicating that the proposed periodic mutation and FHOBL strategies effectively improve the search accuracy of the original algorithm.

Furthermore, IALA exhibits competitive performance on the multimodal, hybrid, and composition functions, such as F13–F24 and F30, where the search landscape is more complex and contains numerous local optima. These results suggest that the proposed strategies enhance population diversity while maintaining efficient exploitation, enabling IALA to escape local optima and obtain better-quality optimal solutions than the compared algorithms.

Table 3 presents the standard deviation (std) on the CEC2017 benchmark suite. A smaller standard deviation indicates better stability and robustness. As shown in Table 3, the proposed IALA achieves lower or highly competitive standard deviations on most benchmark functions, demonstrating its excellent convergence stability and reliable optimization performance.

Compared with ALA, IALA significantly reduces the std on most test functions, indicating that the proposed periodic mutation and FHOBL strategies effectively improve the consistency of the search process while avoiding premature convergence. Although LSHADE achieves slightly lower std on several simple functions, IALA exhibits more stable performance on the more challenging multimodal, hybrid, and composition functions. These results demonstrate that the proposed strategies not only improve optimization accuracy but also enhance the robustness and reliability of the ALA across different optimization problems.

Table 4 reports the mean fitness values obtained by different algorithms on the 30-dimensional CEC2020 benchmark functions. From the overall results, the proposed IALA exhibits strong optimization capability and obtains the lowest mean values on most test functions, such as H2, H3, H4, H5, H7, H9, and H10. In comparison with the original ALA, IALA achieves smaller mean fitness values on the majority of functions, which suggests that the incorporation of periodic mutation and the proposed FHOBL strategy can effectively strengthen the search performance of ALA.

Moreover, IALA performs particularly well on complex hybrid and composition functions, which usually involve irregular landscapes and multiple local optima. Although LSHADE obtains the best results on some functions, such as H1 and H8, IALA still shows better comprehensive performance in terms of optimization accuracy and stability across the whole benchmark set. These findings indicate that the proposed improvement mechanisms help IALA maintain a better trade-off between global exploration and local exploitation, thereby leading to more reliable optimization results on the CEC2020benchmark.

Table 5 reports the best fitness values of all compared algorithms on the 30-dimensional CEC2020 benchmark suite. As can be observed, the proposed IALA achieves the best results on most functions, including H2, H3, H4, H6, H7, H9, and H10. Compared with the ALA, IALA consistently obtains lower best fitness values on the majority of test functions, indicating that the periodic mutation and FHOBL strategies effectively improve the search capability of ALA. Although LSHADE achieves the optimal result on H1 and performs comparably on H8, IALA demonstrates superior overall performance across the benchmark suite, particularly on the more challenging functions such as H5 and H7, where it significantly outperforms the other algorithms. These results indicate that the proposed IALA possesses a stronger ability to locate high-quality solutions and maintain excellent optimization performance on complex optimization problems.

Table 6 reports the standard deviation results of all compared algorithms on the 30-dimensional CEC2020 benchmark functions. A smaller standard deviation indicates better stability and robustness. As shown in Table 6, the proposed IALA obtains lower or competitive standard deviation values on most test functions. Compared with ALA, IALA shows smaller standard deviations on 9 out of 10 functions, indicating that the proposed periodic mutation and FHOBL strategies effectively improve the stability of ALA. Although LSHADE, GWO, and UFIA achieve lower standard deviations on several individual functions, IALA performs more stably than most compared algorithms, especially when compared with PSO, DE, and GGO. In particular, IALA obtains the lowest standard deviation on H5 and maintains competitive robustness on H7 and H8, which are relatively complex benchmark functions. These results suggest that the proposed strategies not only enhance the optimization accuracy of IALA but also improve its convergence reliability on the CEC2020 benchmark suite.

### 3.3. Statistical Analysis

#### 3.3.1. Wilcoxon and Friedman Test

To further verify whether the performance differences among algorithms are statistically significant, the Wilcoxon rank-sum test is employed in this study. In Table 7, “+/=/−“ denotes the number of functions on which IALA performs significantly better than, statistically similar to, or significantly worse than the compared algorithm, respectively.

Table 7 reports the statistical comparison results of IALA against other algorithms on the CEC2017 and CEC2020 benchmark functions with different dimensions. Overall, IALA achieves a clear statistical advantage over most compared algorithms. Compared with ALA, IALA obtains 25/3/1, 25/4/0, and 29/0/0 on the 30-, 50-, and 100-dimensional CEC2017 functions, respectively, and achieves 8/2/0, 9/1/0, and 10/0/0 on the corresponding CEC2020 functions. These results indicate that the proposed periodic mutation and FHOBL strategies significantly improve the optimization performance of ALA.

Furthermore, IALA shows strong superiority over PSO, SSLO, DE, UFIA, and GWO in most cases, especially in high-dimensional optimization problems. For example, on the 100-dimensional CEC2017 benchmark, IALA significantly outperforms ALA, PSO, and UFIA on almost all functions. On the CEC2020 benchmark, IALA also obtains dominant results against most competitors, such as 10/0/0 against UFIA in all tested dimensions. Although LSHADE shows competitive performance on several functions, IALA still achieves more significant wins than losses in most dimensions. Therefore, the statistical results further confirm that IALA has better robustness, scalability, and overall optimization capability than the compared algorithms.

#### 3.3.2. Friedman Analysis

The Friedman test is a non-parametric statistical method commonly used to compare the performance differences among multiple algorithms over multiple benchmark functions. Unlike parametric tests, it does not require the data to follow a normal distribution, making it suitable for analyzing optimization results. In this study, the Friedman test is employed to evaluate whether there are statistically significant differences among the compared algorithms on CEC2017 and CEC2020 benchmark functions with different dimensions.

As shown in Table 8, the Friedman *p*-values on all test cases are far smaller than 0.001, including 2017-30, 2017-50, 2017-100, 2020-30, 2020-50, and 2020-100. This indicates that the performance differences among the compared algorithms are statistically significant at the *p* < 0.001 level. Therefore, the null hypothesis that all algorithms have equivalent performance can be rejected. These results demonstrate that the compared algorithms exhibit significant differences in optimization performance across different benchmark suites and dimensions. Combined with the Wilcoxon rank-sum results in Table 7, it can be further concluded that the proposed IALA shows statistically reliable advantages over most competing algorithms, confirming the effectiveness of the periodic mutation and FHOBL strategies.

### 3.4. Scalability Analysis

**Figure 4:**
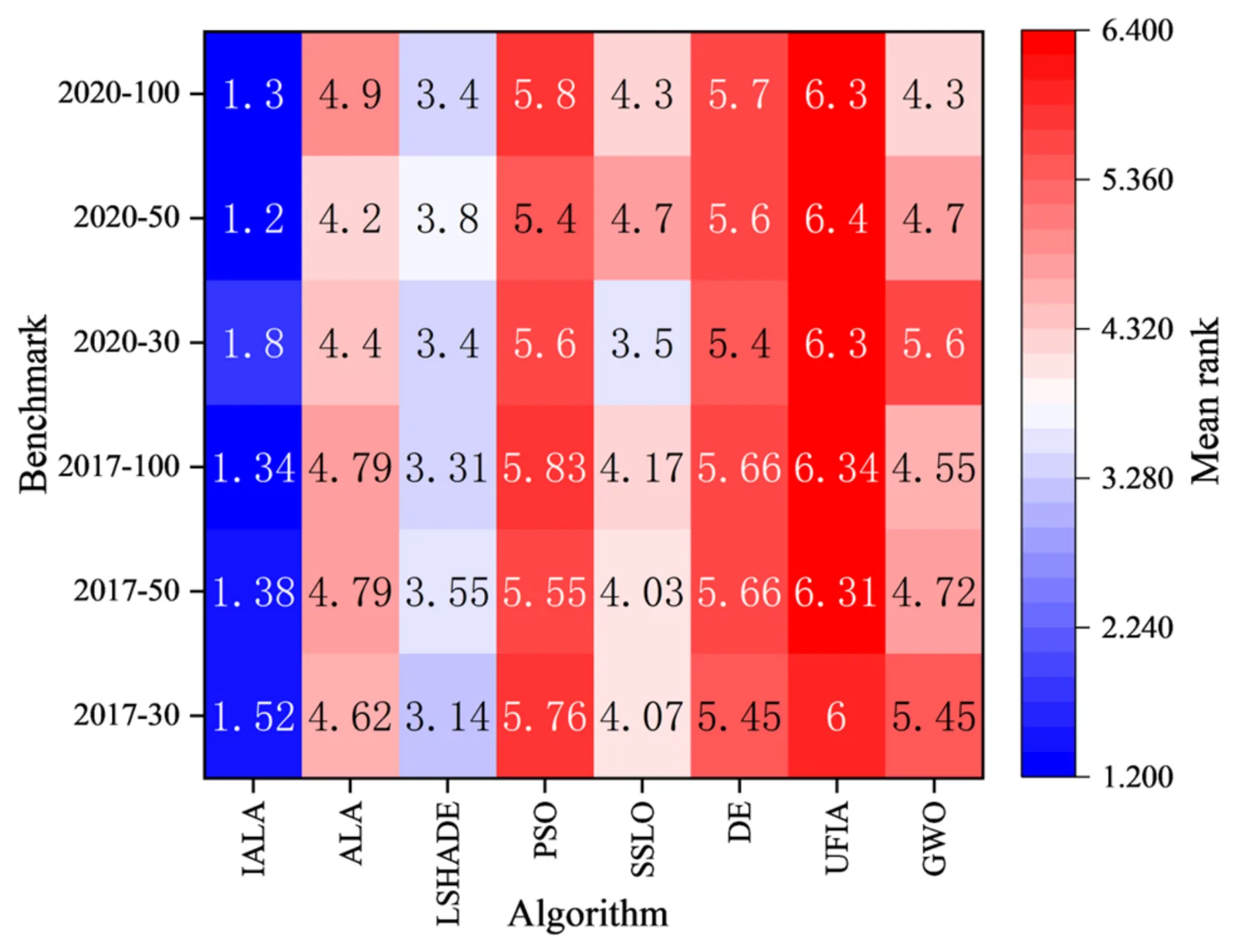
Mean rank heatmap of all algorithms on CEC2017 and CEC2020 benchmark functions with different dimensions.

Figure 4 shows the mean rank heatmap of all algorithms on CEC2017 and CEC2020 benchmark functions with different dimensions. In this heatmap, a smaller mean rank indicates better overall performance, and the color gradually changes from blue to red as the rank becomes worse.

As shown in Figure 4, the proposed IALA obtains the lowest mean rank in all test cases, including 2017-30, 2017-50, 2017-100, 2020-30, 2020-50, and 2020-100. Its mean rank ranges from 1.2 to 1.8, which is clearly lower than those of the other algorithms. This demonstrates that IALA has the best overall optimization performance across different benchmark suites and dimensions.

Compared with ALA, IALA shows a significant improvement in all cases. For example, the mean rank of ALA is mostly around 4.2–4.9, while IALA remains close to the first rank. This result indicates that the proposed periodic mutation and FHOBL strategies effectively enhance the search ability and robustness of ALA. Among the other compared algorithms, LSHADE and SSLO show relatively competitive performance, whereas PSO, DE, UFIA, and GWO generally obtain higher mean ranks. In particular, UFIA presents the highest ranks in most cases, indicating weaker overall performance on these benchmark functions.

Overall, the heatmap visually confirms the statistical superiority of IALA. The consistently dark-blue color of IALA across all dimensions suggests that the proposed algorithm maintains strong robustness and scalability when solving both CEC2017 and CEC2020 benchmark problems.

**Figure 5:**
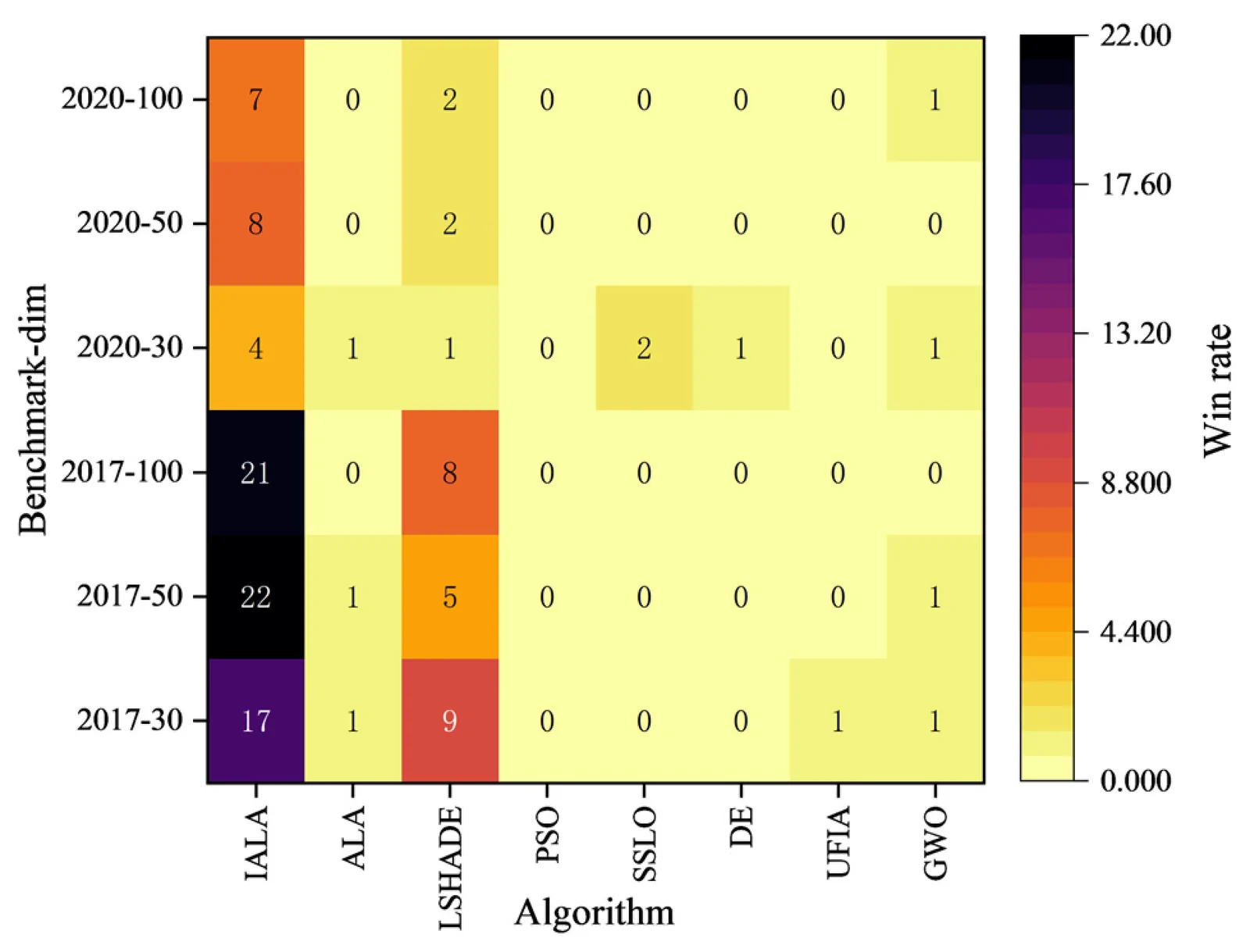
Heatmap of the number of first-rank results obtained by each algorithm on CEC2017 and CEC2020 benchmark functions with different dimensions.

Figure 5 illustrates the number of first-rank results obtained by each algorithm on the CEC2017 and CEC2020 benchmark functions with different dimensions. A larger value indicates that the algorithm achieves the best performance on more test functions.

As shown in Figure 5, the proposed IALA obtains the largest number of first-rank results in almost all benchmark settings. For the CEC2017 test suite, IALA achieves 17, 22, and 21 first-place results on 30-, 50-, and 100-dimensional problems, respectively, which is significantly higher than the other compared algorithms. In particular, LSHADE ranks second on CEC2017, obtaining 9, 5, and 8 wins in the corresponding dimensions, but it is still clearly inferior to IALA.

For the CEC2020 benchmark functions, IALA also shows strong competitiveness. It obtains 4, 8, and 7 first-place results on 30-, 50-, and 100-dimensional problems, respectively. Although LSHADE, SSLO, DE, and GWO achieve the best results on a few individual functions, their overall number of wins is much lower than that of IALA. This indicates that IALA maintains strong optimization capability across different benchmark suites and dimensions.

Compared with ALA, IALA obtains a much larger number of best results, demonstrating that the proposed periodic mutation and FHOBL strategies effectively improve the global search ability and solution quality of ALA. Overall, Figure 4 further confirms the superior robustness, scalability, and comprehensive optimization performance of the proposed IALA.

### 3.5. Representative Convergence Curves

**Figure 6:**
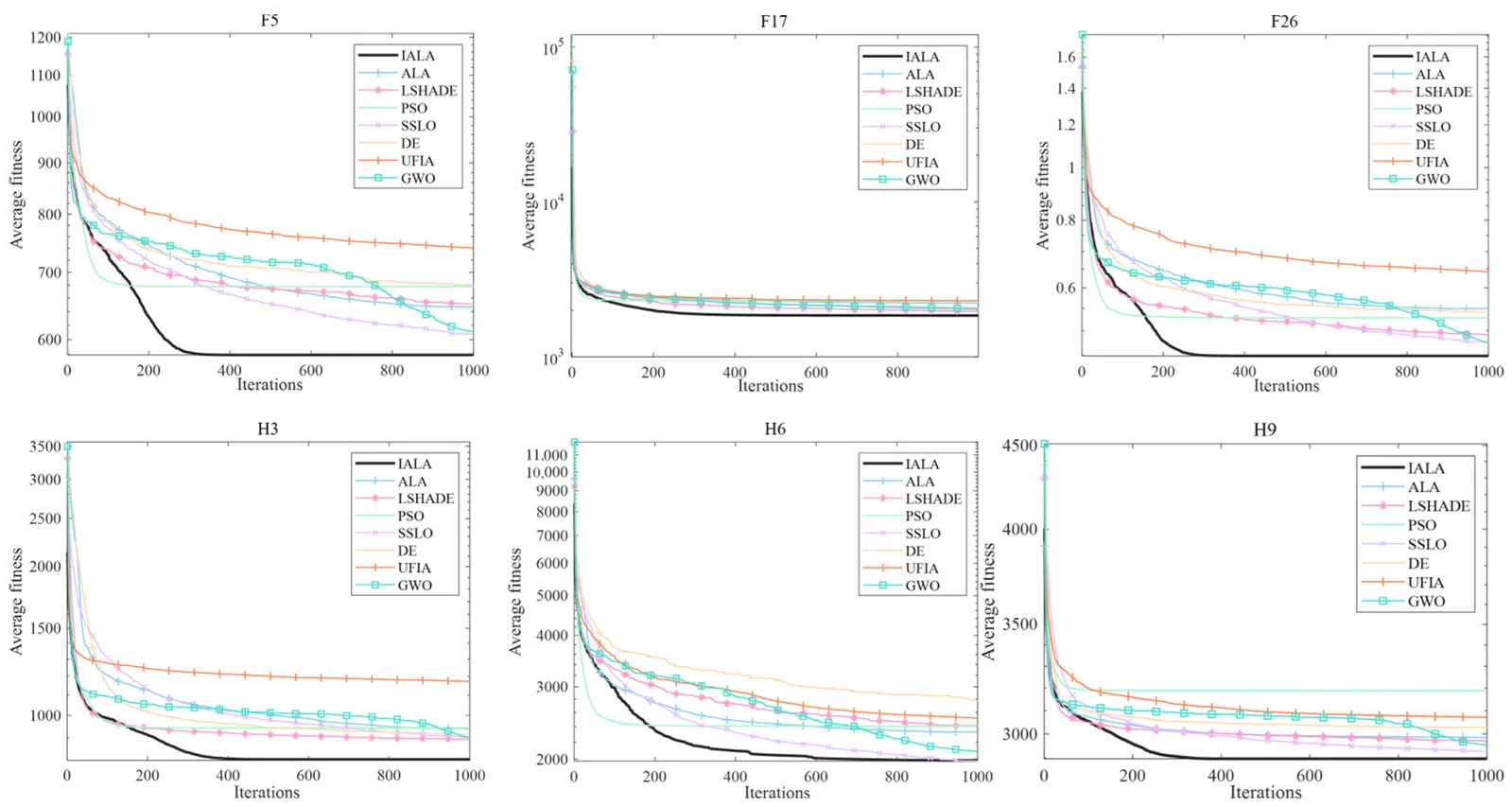
Representative convergence curves of all algorithms on selected 30-dimensional CEC2017 and CEC2020 benchmark functions.

Figure 6 presents several representative convergence curves selected from the 30-dimensional CEC2017 and CEC2020 benchmark functions, where F5, F17, and F26 are selected from CEC2017, and H3, H6, and H9 are selected from CEC2020. These curves are used to illustrate the convergence behavior of different algorithms without displaying all benchmark functions.

As shown in Figure 6, the proposed IALA generally exhibits faster convergence speed and better final fitness values than the compared algorithms. On the CEC2017 functions, especially F5 and F26, IALA rapidly reduces the fitness value in the early stage and reaches a lower convergence level than the other algorithms. This indicates that the proposed periodic mutation and FHOBL strategies can effectively enhance both global exploration and local exploitation. For F17, although several algorithms show similar convergence trends after the early iterations, IALA still maintains competitive final accuracy.

For the CEC2020 functions, IALA also shows strong convergence performance. On H3 and H6, IALA achieves a faster decline and obtains better final fitness values, demonstrating its ability to handle complex search landscapes. On H9, the differences among algorithms become smaller in the later stage, but IALA still maintains stable and competitive convergence behavior. Overall, these representative convergence curves further verify that IALA has better convergence speed, optimization accuracy, and stability than ALA and most other compared algorithms.

## 4. Application to *Dendrobium huoshanense* Polysaccharide Prediction

In this application, the prediction task was formulated as a nonlinear regression from 119 NIR spectral variables to a single reference polysaccharide-content value. Location, sampling time, and temperature were not used as explanatory variables. To reduce the dependence of model performance on a particular training–test partition, five independent repeated random-split experiments were conducted. In each repetition, the complete dataset of 157 samples was randomly divided into a training set and a test set at a ratio of 3:1, resulting in 118 training samples and 39 test samples. Five consecutive random seeds were used to generate five independent data partitioning schemes. In each experimental run, all models under comparison utilized the same partitioning scheme to ensure a fair comparison. For each partition, normalization parameters were estimated solely based on the corresponding training set and applied directly to the test set, thereby preventing information leakage. Prediction performance was evaluated using R^2^, RMSE, MSE, MAPE, and MAE metrics, and the final results are presented as the mean and standard deviation across the five random partitioning experiments.

**Figure 7:**
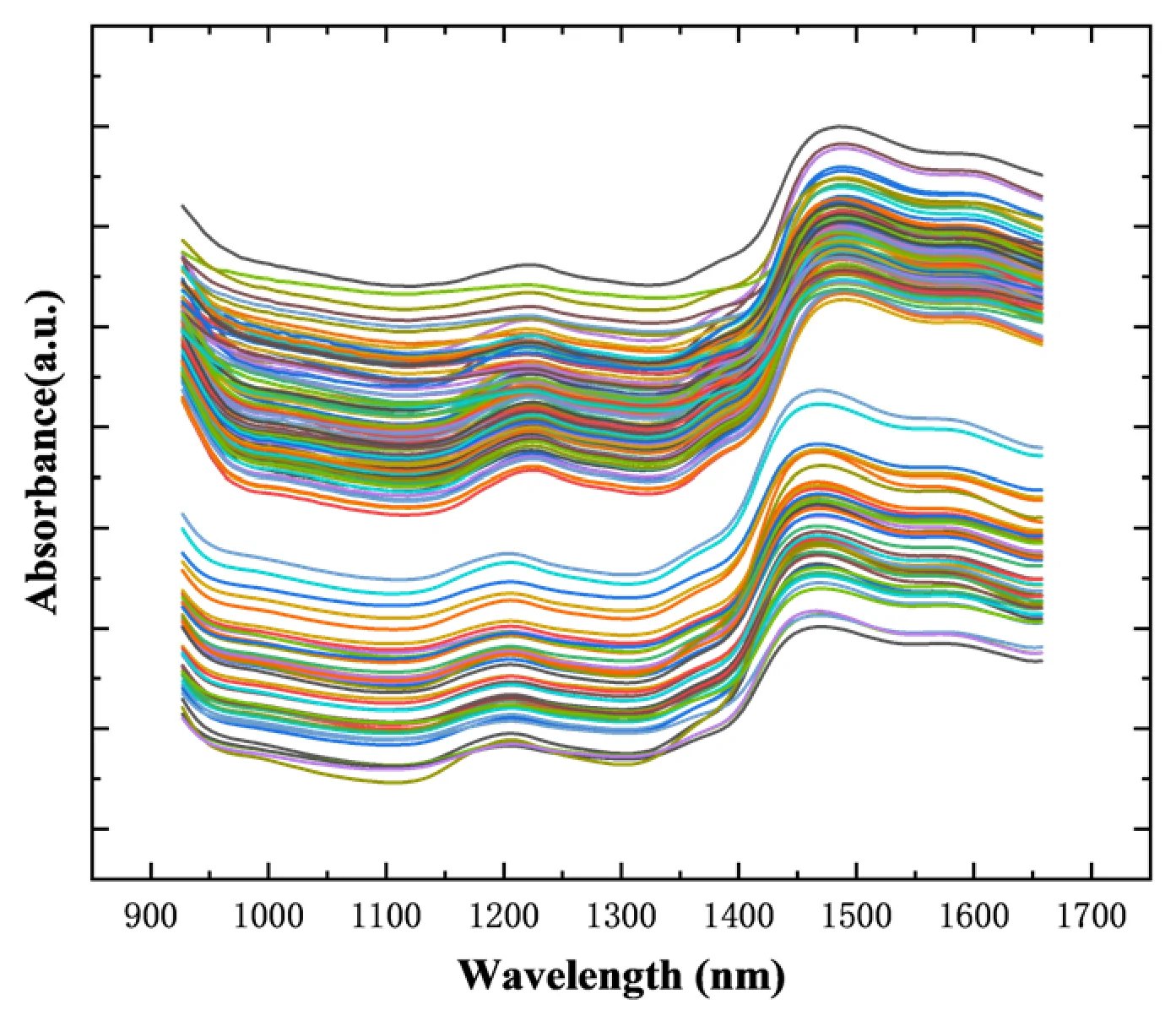
The near-infrared spectral data of *Dendrobium* samples.

Figure 7 shows the near-infrared spectral data of *Dendrobium* samples. The spectral curves of different samples exhibit similar overall trends, indicating that the samples share comparable chemical composition characteristics. Meanwhile, variations in absorbance among samples reflect differences in polysaccharides, moisture, proteins, and other organic components. The absorption features in the near-infrared region are mainly related to the overtones and combination vibrations of O–H, C–H, and N–H bonds. Therefore, the obtained spectral data provide useful information for predicting the polysaccharide content of *Dendrobium*. The modeling dataset contained 157 samples. Each sample was represented by 119 spectral variables, and the final column contained the corresponding reference polysaccharide content. All 119 spectral variables were used as model inputs; no additional wavelength-selection, dimensionality-reduction, or feature-extraction algorithm was applied in this study. After the samples were divided into training and test sets, the input variables were normalized to the range [0, 1] using MATLAB’s mapminmax function. Importantly, the minimum and maximum values were estimated exclusively from the training set, and the same transformation parameters were subsequently applied to the test set. The training target values were normalized in the same manner, and the model predictions were transformed back to the original scale before calculating R^2^, RMSE, MSE, MAPE, and MAE. This procedure prevented information from the test set from entering the model-training process.

Table 9 compares the mean prediction performance of the standard BP neural network and BP models optimized by different algorithms. The evaluation indices include R^2^, RMSE, MSE, MAPE, and MAE. In general, a larger R^2^ and smaller RMSE, MSE, MAPE, and MAE indicate better prediction performance.

As shown in Table 9, the traditional BP model obtains an R^2^ value of 0.8242, which is lower than all optimization-based BP models, indicating that the BP network has limited prediction accuracy. After introducing metaheuristic algorithms to optimize the BP neural network, the prediction performance is significantly improved. Among all models, IALA-BP achieves the highest R^2^ value of 0.8731, as well as the lowest RMSE of 2.1581 and MSE of 4.6683, demonstrating its good fitting ability and prediction accuracy.

Compared with the ALA-BP model, IALA-BP improves R^2^ from 0.8579 to 0.8731, while reducing RMSE from 2.26151 to 2.1581 and MSE from 5.2244 to 4.6683. This indicates that the proposed periodic mutation and FHOBL strategies effectively enhance the optimization ability of ALA and help the BP network obtain better initial weights and thresholds. In terms of MAE, IALA-BP obtains the lowest value of 1.6925, while ALA-BP achieves a value of 1.7727. Therefore, considering the overall evaluation results, IALA-BP exhibits the best comprehensive prediction performance and is more suitable for predicting the polysaccharide content of *Dendrobium*.

Table 10 presents the standard deviations of the test-set performance metrics obtained by different models over five independent runs. A smaller standard deviation indicates lower performance fluctuation and better stability and reproducibility. IALA-BP achieves the lowest standard deviations for all five metrics, with values of 0.0138, 0.1166, 0.5065, 0.3431, and 0.0988 for R^2^, RMSE, MSE, MAPE, and MAE, respectively. These results demonstrate that IALA-BP provides the most stable performance among all the evaluated models.

Compared with the conventional BP model, IALA-BP reduces the standard deviations of R^2^, RMSE, MSE, MAPE, and MAE by approximately 79.68%, 75.31%, 79.72%, 74.28%, and 74.87%, respectively. This substantial reduction indicates that IALA effectively decreases the sensitivity of the BP model to random initialization and variations in the training process. Among the remaining optimized models, PSO-BP exhibits the second-best overall stability, followed by GWO-BP, SSLO-BP, and UFIA-BP. In contrast, DE-BP produces relatively large standard deviations for R^2^, RMSE, MSE, and MAE, while LSHADE-BP has the largest MAPE standard deviation, indicating greater variability across repeated experiments.

Overall, IALA-BP exhibits superior robustness and reproducibility compared with the other models. It should be noted that standard deviation primarily reflects model stability rather than predictive accuracy. Therefore, these results should be interpreted together with the mean performance metrics. If IALA-BP also achieves a higher mean R^2^ and lower mean RMSE, MSE, MAPE, and MAE, it can be concluded that the proposed model provides advantages in both predictive accuracy and stability.

**Figure 8:**
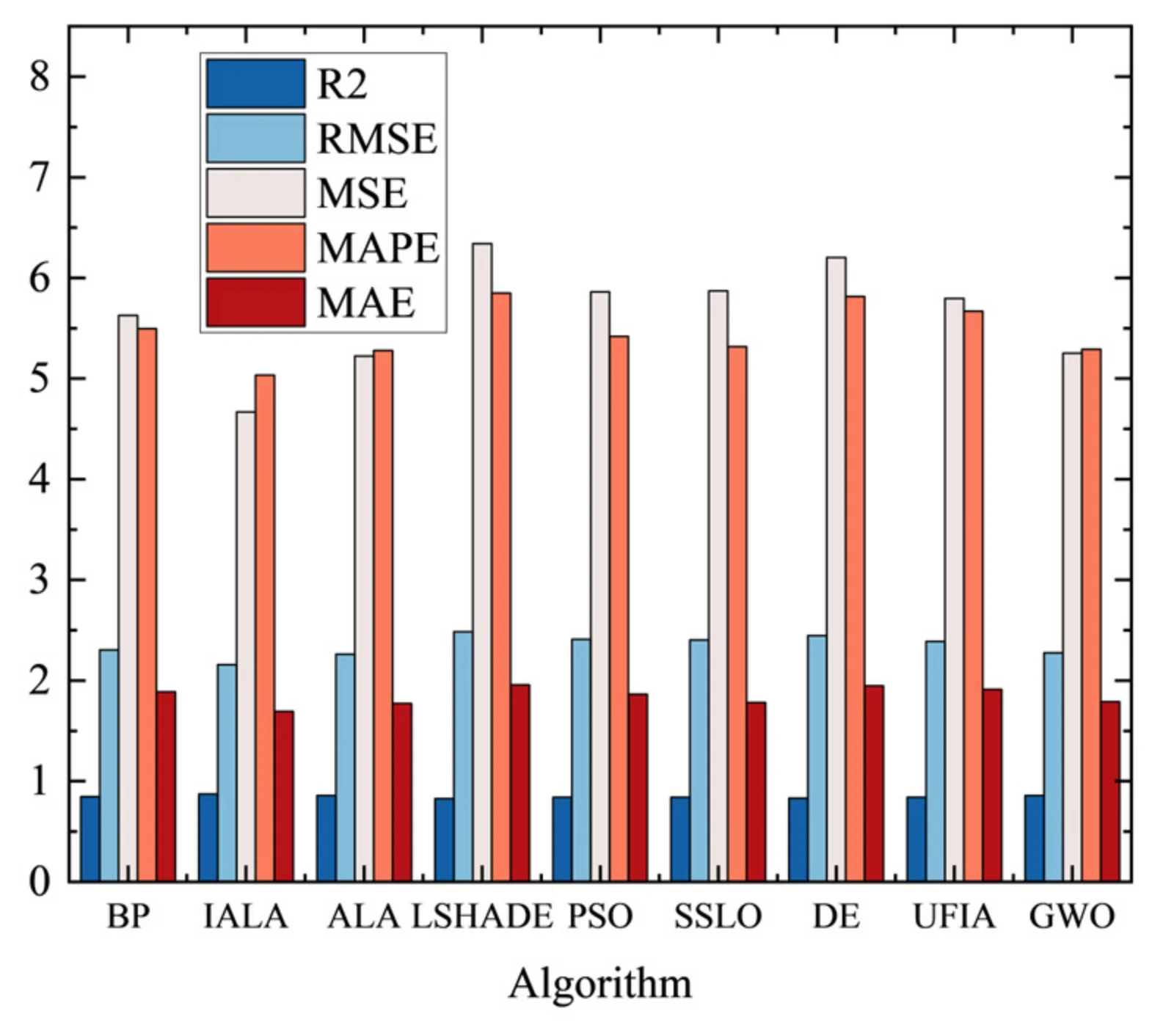
Bar chart of mean prediction performance comparison of BP and different optimization-based BP models.

Figure 8 shows the bar chart corresponding to the prediction results in Table 9. The figure visually compares the performance of BP and different optimization-based BP models in terms of R^2^, RMSE, MSE, MAPE, and MAE. Overall, the optimized BP models perform better than the BP model, indicating that metaheuristic algorithms can effectively improve the prediction accuracy of BP neural networks.

Among all models, IALA-BP achieves the highest R^2^ and the lowest RMSE, MSE and MAE demonstrating its stronger fitting ability and prediction accuracy. Compared with ALA-BP, the performance of IALA-BP is improved, which further verifies the effectiveness of the proposed periodic mutation and FHOBL strategies. Therefore, the results in Figure 8 further confirm that IALA-BP has better comprehensive prediction capability and stability for *Dendrobium* polysaccharide content prediction than the standard BP and other optimization-based BP models.

**Figure 9:**
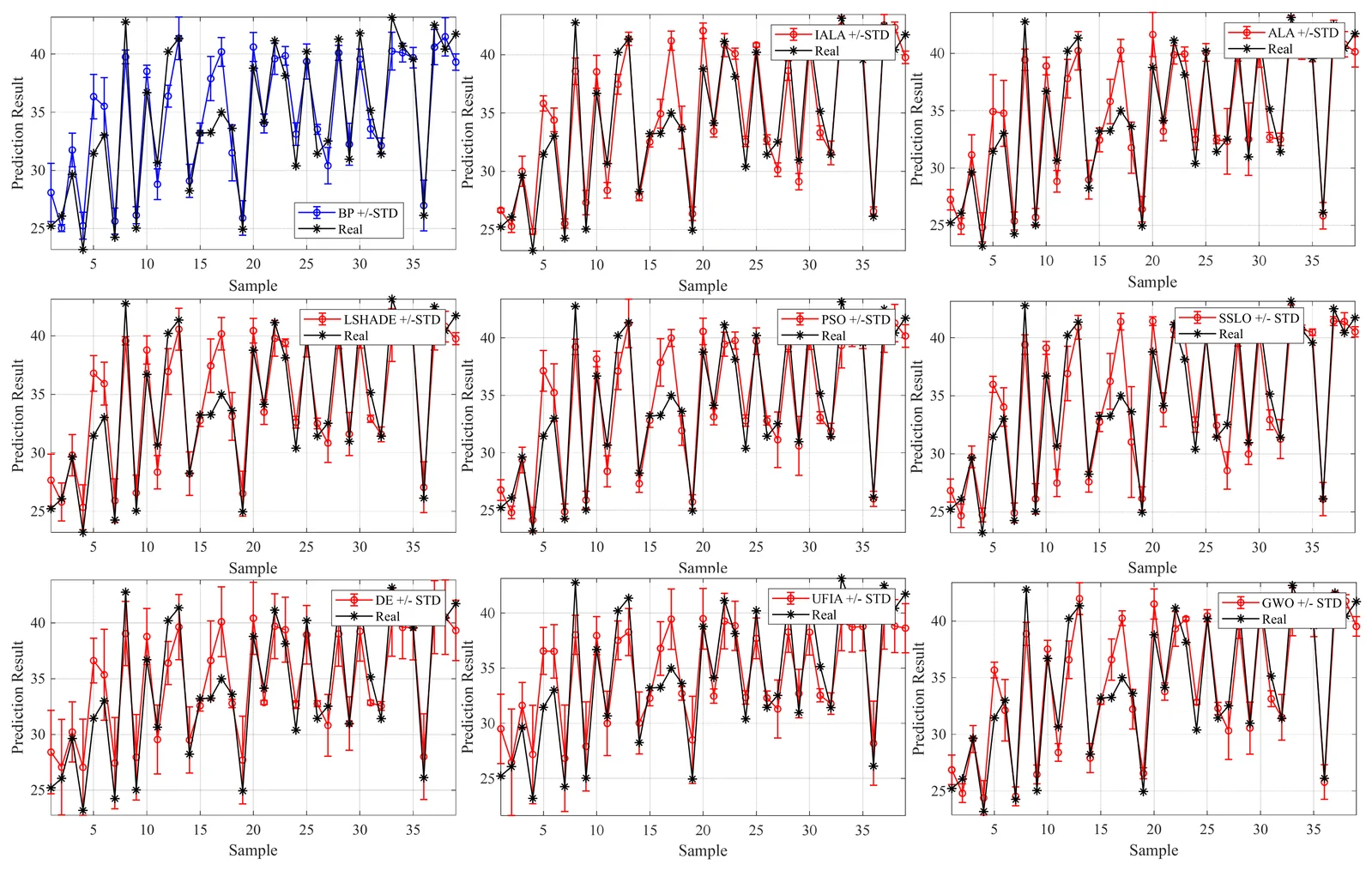
Comparison between measured and predicted values of different BP-based models on the test set.

Figure 9 shows the comparison between the measured values and predicted values of different BP-based models on the test set. The black curve represents the actual polysaccharide content, while the colored curves represent the prediction results of different models. Overall, all optimized BP models can follow the variation trend of the measured values to a certain extent, indicating that the BP neural network has applicability in *Dendrobium* polysaccharide content prediction.

Compared with the BP model, the prediction curves of the optimization-based BP models are generally closer to the real values, which demonstrates that metaheuristic algorithms can effectively improve the prediction performance of BP by optimizing its initial weights and thresholds. Among them, the IALA-BP model shows the best fitting performance, with its predicted values highly consistent with the real values in most samples. This indicates that the proposed IALA can effectively enhance the learning ability and generalization performance of BP.

In contrast, the prediction curves of ALA-BP, DE-BP, and some other models show larger deviations at several sample points, especially in regions with sharp fluctuations. Although PSO-BP and GWO-BP also exhibit good prediction trends, their local prediction errors are still slightly larger than those of IALA-BP. Therefore, Figure 9 further confirms that IALA-BP has better prediction accuracy and stability for *Dendrobium* polysaccharide content prediction.

**Figure 10:**
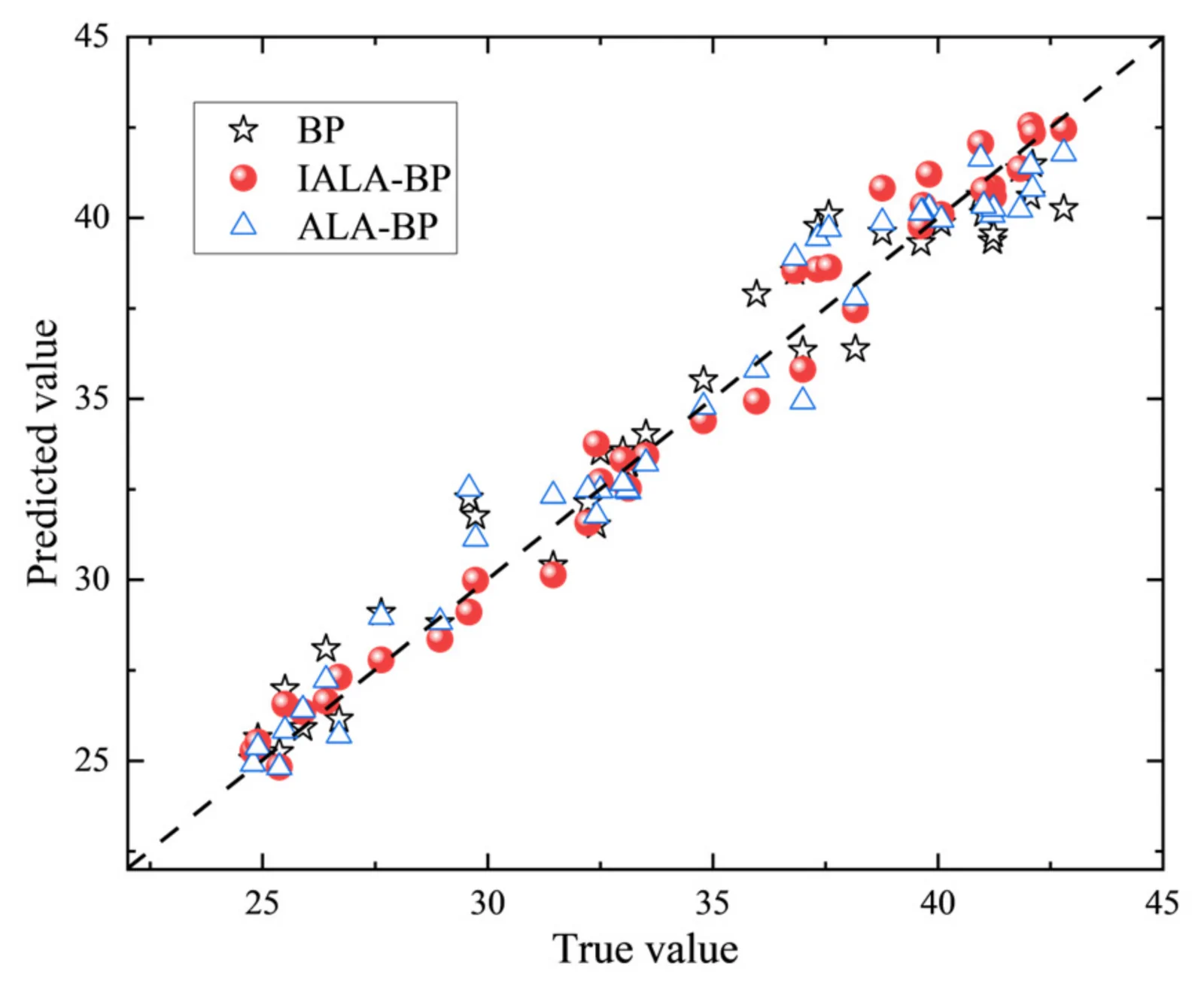
Scatter plot of measured and predicted polysaccharide contents using BP, ALA-BP, and IALA-BP models.

Figure 10 shows the scatter comparison between the measured polysaccharide content and the predicted values obtained by BP, ALA-BP, and IALA-BP models. The horizontal axis represents the true polysaccharide content, while the vertical axis represents the predicted value. In general, the closer the scattered points are to the diagonal reference line, the better the prediction accuracy of the model.

As shown in Figure 10, the prediction results of the three models show a positive correlation with the real values, indicating that BP-based models can effectively capture the relationship between near-infrared spectral features and *Dendrobium* polysaccharide content. However, compared with the BP model and ALA-BP model, the scattered points of IALA-BP are more concentrated around the diagonal line, suggesting that its predicted values are more consistent with the measured values.

In particular, when the polysaccharide content is relatively high, the BP model shows relatively larger prediction deviations, while ALA-BP improves the fitting performance to some extent. The proposed IALA-BP further reduces the deviation between predicted and measured values, demonstrating better prediction accuracy and generalization ability. This result is consistent with the quantitative evaluation results in Table 9, where IALA-BP achieves the highest R^2^ and the lowest RMSE and MSE.

Overall, Figure 10 further verifies that the proposed IALA optimization strategy can effectively improve the prediction performance of the BP neural network for *Dendrobium* polysaccharide content.

## 5. Conclusions

In this study, an improved Artificial Lemming Algorithm, named IALA, was proposed by introducing periodic mutation and FHOBL strategies. Benchmark results on CEC2017 and CEC2020 show that IALA has better optimization accuracy, stability, and robustness than the ALA and most compared algorithms. The Wilcoxon rank-sum test, Friedman test, heatmaps, and convergence curves further confirm the effectiveness of the proposed improvement strategies.

Furthermore, IALA was applied to optimize the initial weights and thresholds of BP neural networks for *Dendrobium* polysaccharide content prediction. The proposed IALA-BP model achieved the highest R^2^ and the lowest RMSE and MSE among all compared models, indicating its superior fitting ability and prediction accuracy. The comparison between measured and predicted values also shows that IALA-BP can better capture the nonlinear relationship between near-infrared spectral data and polysaccharide content.

Overall, the proposed IALA-BP model provides an effective method for *Dendrobium* polysaccharide content prediction. Nevertheless, the present study was based on a relatively limited dataset of 157 samples, and the repeated random-split validation was conducted within the same overall sample population. In future work, more *Dendrobium* samples from different origins, growth years, and processing conditions will be collected to further improve the robustness and generalization ability of the model. Meanwhile, various spectral preprocessing and feature selection methods will be investigated to reduce spectral noise, eliminate redundant variables, and identify key wavelengths related to polysaccharide content. In addition, the proposed model will be compared with more advanced machine learning and deep learning methods to further evaluate its applicability. The prediction of other active components in *Dendrobium* will also be explored to support more comprehensive and rapid quality assessment.

## Figures and Tables

**Table 1:** Mean fitness values of all algorithms on 30-dimensional CEC2017 benchmark functions.

	IALA	ALA	LSHADE	PSO	SSLO	DE	UFIA	GWO
F1	4806.8	5,613,726.9	100.1	1,570,473,572.4	11,165,029.1	49,916.6	313,241,813.4	2,186,848,109.8
F3	708.0	19,933.7	57,151.9	39,879.6	108,093.8	151,893.7	37,766.3	50,298.6
F4	481.0	513.9	482.1	751.3	534.7	501.8	600.4	651.9
F5	579.4	646.9	650.3	677.7	607.0	679.6	740.4	611.3
F6	601.7	625.3	600.0	639.6	602.6	600.0	647.2	609.7
F7	825.9	926.4	892.4	925.7	902.9	914.0	1145.6	917.2
F8	871.0	951.7	948.7	933.3	911.0	983.7	994.7	899.1
F9	1317.7	3209.1	901.7	3704.9	1744.5	1656.6	5309.2	2356.5
F10	4833.3	5462.6	7692.8	4894.0	4992.0	7112.9	7066.4	4944.9
F11	1251.2	1475.3	1264.2	1322.3	5325.7	4985.9	1559.9	3125.5
F12	196,664.6	2,175,517.6	64,021.4	38,921,674.8	5,544,324.9	21,660,469.4	3,108,523.1	89,804,089.9
F13	15,991.3	46,290.2	25,220.6	500,643.6	200,584.1	1,331,010.6	15,401.4	7,094,898.5
F14	1632.3	1579.2	1578.7	13,119.9	94,060.7	239,960.6	31,090.7	678,919.4
F15	3873.2	12,894.4	2069.1	10,515.1	23,670.5	197,986.6	20,279.2	2,487,456.8
F16	2343.4	2698.7	2746.9	2811.4	2658.9	2770.2	2991.9	2594.1
F17	1847.8	2226.9	1965.0	2301.0	2017.4	2087.1	2295.9	2043.1
F18	26,057.8	12,532.0	166,493.2	167,734.0	670,895.2	1,834,722.2	368,671.9	1,966,057.7
F19	8015.6	15,333.5	2073.6	31,385.3	35,406.0	223,311.5	43,895.7	8,095,494.0
F20	2258.7	2520.3	2306.6	2598.9	2377.6	2396.5	2621.3	2475.1
F21	2532.0	3027.4	3373.9	3254.9	2783.2	3282.1	2556.9	3305.0
F22	12,672.7	14,592.1	18,878.7	13,824.8	12,994.0	18,018.7	18,220.4	12,041.7
F23	3469.2	4387.1	4279.7	5341.1	3738.0	4478.0	5031.4	3922.4
F24	3529.8	4245.3	4259.9	4422.4	3595.6	5023.3	4083.8	4089.4
F25	2880.4	2898.2	2880.0	2968.4	2917.8	2882.0	2961.3	2996.6
F26	4493.2	5493.8	4909.1	5280.5	4762.1	5396.4	6419.1	4760.5
F27	3227.3	3248.9	3219.2	3303.8	3232.7	3222.6	3346.6	3270.3
F28	3198.1	3712.7	3223.9	3417.5	3298.1	3284.1	3377.9	3463.7
F29	3654.1	4063.3	3806.7	4265.3	3773.1	3978.6	4361.8	3894.8
F30	8510.1	92,262.5	29,519.7	253,097.0	73,469.5	176,752.0	117,485.7	7,029,468.7

**Table 2:** Best fitness values of all algorithms on 30-dimensional CEC2017 benchmark functions.

	IALA	ALA	LSHADE	PSO	SSLO	DE	UFIA	GWO
F1	106.8	1,111,482.6	100.0	1,728,244.3	3,820,892.8	15,565.9	1,507,939.5	163,091,849.3
F3	355.5	8357.0	35,222.7	5659.9	79,425.0	82,613.2	22,967.5	24,014.1
F4	400.2	475.0	400.0	488.8	513.1	490.9	486.9	517.9
F5	547.8	598.0	616.9	592.9	589.5	654.9	664.8	563.1
F6	600.1	604.0	600.0	623.8	602.0	600.0	633.4	605.3
F7	783.5	853.6	860.4	839.8	867.4	895.9	1002.8	842.2
F8	837.8	893.3	929.9	882.6	884.0	962.4	938.1	856.9
F9	987.1	1372.0	900.1	2112.2	1342.5	1209.6	3547.2	1181.3
F10	3726.0	4099.1	6929.5	3981.8	4351.0	6592.4	5901.6	3361.7
F11	1169.1	1316.0	1177.4	1187.6	2815.6	3290.6	1278.8	1380.0
F12	40,762.2	445,384.9	8146.5	122,440.3	1,541,470.6	9,471,801.2	258,257.3	3,477,460.6
F13	1371.1	3615.3	7366.9	6255.7	34,151.3	196,665.5	2930.1	42,596.5
F14	1495.8	1523.6	1526.5	2248.5	22,460.7	36,008.0	2163.3	3522.1
F15	1564.5	2037.6	1739.9	2222.1	4272.8	54,567.4	2255.8	17,442.1
F16	1847.9	2077.3	2426.9	2393.1	2425.7	2517.0	2292.1	2026.0
F17	1741.0	1837.4	1850.8	1771.8	1837.6	1914.7	1970.4	1826.6
F18	5707.7	2488.1	74,063.6	57,667.7	97,379.8	549,669.5	66,269.6	186,277.1
F19	2001.1	2053.6	1973.8	1994.8	2922.8	60,442.1	2339.0	23,460.8
F20	2025.0	2329.2	2154.2	2298.1	2230.6	2188.1	2395.3	2221.2
F21	2200.0	2216.3	2200.0	2272.0	2289.1	2466.0	2250.0	2285.9
F22	7356.5	9944.4	17,270.6	10,378.8	11,750.7	16,121.4	15,968.4	9331.3
F23	3174.7	3796.6	3904.6	2880.7	3550.1	3364.0	2919.5	3470.3
F24	2600.0	3597.1	2600.0	2616.1	2640.8	4211.0	2624.1	3486.7
F25	2878.6	2881.1	2878.6	2911.3	2893.5	2880.6	2889.4	2916.9
F26	2900.0	2852.4	2800.0	2840.6	4219.8	5198.7	3644.4	4198.0
F27	3212.1	3205.0	3204.8	3230.5	3211.4	3214.5	3272.1	3210.4
F28	3141.2	3236.6	3195.0	3260.9	3268.2	3246.5	3254.2	3323.7
F29	3366.4	3582.4	3562.5	3826.4	3575.4	3716.9	3836.5	3576.7
F30	5570.2	9754.8	10,572.3	21,649.8	35,823.8	88,176.0	7938.8	1,603,044.9

**Table 3:** The standard deviation of all algorithms on 30-dimensional CEC2017 benchmark functions.

	IALA	ALA	LSHADE	PSO	SSLO	DE	UFIA	GWO
F1	5284.6	3,506,115.4	0.1	2,544,085,064.0	3,461,397.1	30,757.7	600,191,067.9	1,306,915,091.8
F3	327.2	6250.5	14,570.6	18,545.8	18,895.9	35,604.8	8256.9	12,726.7
F4	23.3	31.9	31.0	452.2	11.1	10.9	99.9	175.6
F5	20.2	30.3	13.0	36.6	12.4	10.7	36.6	45.4
F6	2.5	13.6	0.0	9.9	0.4	0.0	11.0	2.8
F7	24.5	43.0	15.9	44.5	15.8	12.6	92.7	65.1
F8	20.8	32.4	13.7	28.0	10.3	13.9	36.4	30.3
F9	355.9	1437.1	1.6	1024.3	273.3	317.0	1143.4	802.4
F10	538.7	871.7	302.9	483.3	279.9	281.7	804.4	1524.9
F11	71.9	105.2	53.2	75.4	1506.1	1015.5	378.1	1356.4
F12	204,153.0	2,871,296.5	50,729.4	136,604,214.2	1,923,501.2	9,478,941.9	4,385,363.6	99,223,269.7
F13	12,599.4	41,677.9	19,780.5	1,373,139.2	262,307.5	1,044,590.6	17,441.4	29,368,878.2
F14	103.7	42.1	41.0	8786.1	51,293.5	159,752.0	47,491.9	618,154.7
F15	4312.7	15,624.8	187.3	12,180.4	14,791.0	105,832.0	17,704.9	8,272,366.5
F16	309.2	349.7	181.3	242.5	110.8	132.1	301.0	335.8
F17	82.4	248.7	112.2	270.3	99.1	116.2	175.9	153.9
F18	17,013.8	10,689.3	83,071.1	99,698.5	423,366.4	888,137.0	393,751.3	2,141,694.9
F19	5631.2	16,080.3	80.4	51,327.2	27,443.3	147,270.4	129,157.0	30,310,749.5
F20	119.6	155.4	107.5	175.6	96.9	91.1	112.3	152.1
F21	361.3	741.8	712.5	696.5	500.2	666.6	688.8	488.6
F22	2179.8	2517.9	980.4	1842.2	630.1	953.4	1089.2	2783.4
F23	263.5	294.8	144.0	874.3	114.9	281.7	741.4	244.7
F24	399.3	462.3	421.4	925.0	556.5	258.4	1351.6	512.9
F25	1.3	15.6	0.5	49.6	13.8	1.5	43.2	55.4
F26	438.3	801.3	533.1	1496.2	174.2	111.7	1191.6	342.8
F27	10.8	64.9	12.2	41.7	8.8	4.9	56.5	41.2
F28	23.3	1057.5	21.5	153.7	17.4	24.6	73.4	84.1
F29	142.7	247.2	117.7	259.1	107.6	149.5	383.8	250.1
F30	2654.1	187,279.4	18,982.6	256,578.1	32,167.0	127,139.7	404,919.8	7,481,185.3

**Table 4:** Mean fitness values of all algorithms on 30-dimensional CEC2020 benchmark functions.

	IALA	ALA	LSHADE	PSO	SSLO	DE	UFIA	GWO
H1	4276.4	5,675,364.9	100.1	595,247,781.8	12,641,818.9	62,016.4	206,447,544.7	3,223,993,989.9
H2	4856.6	6043.0	7691.4	4921.0	4850.6	7327.2	7371.9	4850.2
H3	814.8	939.2	893.3	942.8	901.3	916.1	1171.7	903.7
H4	1909.6	1921.6	1914.3	2417.2	1918.6	1917.6	3348.2	6753.1
H5	40,055.5	40,457.9	314,056.1	608,324.5	2,895,473.7	5,862,021.6	753,149.0	3,041,462.6
H6	1985.7	2325.1	2411.0	2404.9	1979.6	2777.9	2516.2	2088.8
H7	12,407.2	10,974.4	33,958.1	134,552.2	489,418.9	1,172,989.2	283,922.5	1,623,832.6
H8	2664.6	6702.1	2630.7	4485.6	2520.9	5834.9	2874.0	5523.8
H9	2897.5	2985.8	2972.1	3188.4	2927.1	3027.4	3071.3	2954.9
H10	2889.9	2904.6	2891.7	2957.3	2917.9	2888.9	2984.5	3025.8

**Table 5:** Best fitness values of all algorithms on 30-dimensional CEC2020 benchmark functions.

	IALA	ALA	LSHADE	PSO	SSLO	DE	UFIA	GWO
H1	106.2	1,436,565.3	100.0	1,585,141.0	7,038,297.5	25,399.7	473,173.8	383,403,005.8
H2	3949.1	4293.5	6778.9	3619.8	4228.4	6561.5	5989.9	3704.8
H3	787.1	855.8	858.5	842.1	868.4	886.7	1023.0	827.2
H4	1904.9	1916.8	1911.3	1930.5	1914.8	1914.6	1985.2	1912.2
H5	10,141.4	5748.7	25,206.2	45,691.5	1,193,429.8	3,081,697.2	110,532.7	242,177.2
H6	1682.3	1867.2	2080.1	1986.5	1809.0	2462.5	1930.7	1803.3
H7	2953.5	4280.6	8851.7	18,649.1	116,243.2	380,170.0	29,408.4	36,710.8
H8	2300.0	2337.4	2300.0	2363.9	2317.1	2803.5	2329.3	2508.4
H9	2847.3	2905.7	2932.5	3047.5	2899.7	2989.9	2957.0	2861.8
H10	2883.5	2890.2	2887.0	2892.1	2907.7	2887.8	2919.2	2938.7

**Table 6:** The standard deviation of all algorithms on 30-dimensional CEC2020 benchmark functions.

	IALA	ALA	LSHADE	PSO	SSLO	DE	UFIA	GWO
H1	5515.7	5,845,849.8	0.1	719,472,287.8	4,018,831.6	26,993.7	407,371,640.7	3,180,909,142.7
H2	727.2	981.5	358.9	688.7	313.4	397.5	796.6	1013.2
H3	17.4	35.1	15.8	41.2	13.8	12.7	78.2	54.9
H4	2.6	3.6	2.0	777.4	2.1	1.4	2047.1	12,441.9
H5	44,896.6	66,676.9	240,858.6	684,913.9	1,250,311.7	1,951,281.7	603,675.8	2,436,133.1
H6	211.4	240.2	170.1	255.4	76.1	165.8	274.4	157.7
H7	19,568.5	16,757.1	31,852.6	133,801.6	331,592.1	645,200.8	255,462.1	2,260,700.1
H8	1126.2	1649.0	1477.6	2221.7	872.9	2100.6	1594.1	2608.7
H9	29.7	36.4	18.6	89.7	13.0	11.4	73.6	63.2
H10	11.6	19.0	11.1	33.4	8.5	1.3	44.3	101.3

**Table 7:** Wilcoxon rank-sum test results on CEC2017 and CEC2020 benchmark functions with different dimensions.

Compared Algorithms	2017-30 (+/=/−)	2017-50 (+/=/−)	2017-100 (+/=/−)	2020-30 (+/=/−)	2020-50 (+/=/−)	2020-100 (+/=/−)
IALA VS ALA	25/3/1	25/4/0	29/0/0	8/2/0	9/1/0	10/0/0
IALA VS LSHADE	16/6/7	19/6/4	18/5/6	7/1/2	8/2/0	7/1/2
IALA VS PSO	25/4/0	28/1/0	29/0/0	9/1/0	8/2/0	10/0/0
IALA VS SSLO	26/3/0	27/1/1	28/0/1	7/2/1	9/1/0	10/0/0
IALA VS DE	27/1/1	27/1/1	28/0/1	9/0/1	10/0/0	10/0/0
IALA VS UFIA	25/4/0	28/1/0	29/0/0	10/0/0	10/0/0	10/0/0
IALA VS GWO	26/3/0	27/2/0	26/3/0	8/2/0	7/3/0	7/3/0

**Table 8:** Friedman test results of all algorithms on CEC2017 and CEC2020 benchmark functions with different dimensions.

	2017-30	2017-50	2017-100	2020-30	2020-50	2020-100
Friedman *p*-value	1.27 × 10^−14^	9.64 × 10^−15^	4.38 × 10^−16^	3.88 × 10^−4^	1.69 × 10^−4^	9.10 × 10^−5^
Significance	*p* < 0.001	*p* < 0.001	*p* < 0.001	*p* < 0.001	*p* < 0.001	*p* < 0.001

**Table 9:** Mean prediction performance comparison of BP and different optimization-based BP models.

	R^2^	RMSE	MSE	MAPE	MAE
BP	0.8470	2.3046	5.6284	5.4946	1.8864
IALA-BP	0.8731	2.1581	4.6683	5.0329	1.6925
ALA-BP	0.8579	2.2615	5.2244	5.2786	1.7727
LSHADE-BP	0.8276	2.4847	6.3403	5.8491	1.9564
PSO-BP	0.8407	2.4104	5.8603	5.4176	1.8621
SSLO-BP	0.8404	2.4042	5.8713	5.3162	1.7818
DE-BP	0.8314	2.4467	6.2036	5.8158	1.9470
UFIA-BP	0.8425	2.3891	5.7955	5.6685	1.9119
GWO-BP	0.8573	2.2758	5.2514	5.2917	1.7883

**Table 10:** Standard deviations of test-set performance metrics for different BP-based prediction models over five independent runs.

	R^2^	RMSE	MSE	MAPE	MAE
BP	0.0913	0.6298	3.3615	1.7449	0.5951
IALA-BP	0.0138	0.1166	0.5065	0.3431	0.0988
ALA-BP	0.0497	0.3707	1.8300	0.8052	0.2794
LSHADE-BP	0.0669	0.4558	2.4598	1.6012	0.4507
PSO-BP	0.0330	0.2499	1.2150	0.6133	0.2198
SSLO-BP	0.0439	0.3372	1.6151	0.7243	0.2243
DE-BP	0.0736	0.5210	2.7065	1.3974	0.4545
UFIA-BP	0.0439	0.3312	1.6141	0.8040	0.2979
GWO-BP	0.0363	0.3001	1.3354	0.7567	0.2468

## Data Availability

The original contributions presented in this study are included in the article. Further inquiries can be directed to the corresponding author.
